# Congenital hypofibrinogenemia in pregnancy: a report of 11 cases

**DOI:** 10.1097/MBC.0000000000000676

**Published:** 2017-11-24

**Authors:** He Cai, Meiying Liang, Jingjing Yang, Xiaohui Zhang

**Affiliations:** a Obstetrics and Gynecology Department; b Division of Hematology, Peking University People's Hospital, Beijing, China

**Keywords:** complication, fibrinogen replacement, hypofibrinogenemia, pregnancy

## Abstract

To investigate the obstetrical outcomes and plasma concentrations of fibrinogen (FIB) in patients with congenital hypofibrinogenemia in pregnancy, 11 cases with hypofibrinogenemia in pregnancy were analyzed retrospectively. The demographic database, bleeding tendency, plasma levels of FIB throughout pregnancy, peripartum management, as well as, maternal and neonatal outcomes were assessed. FIB levels in our patients remained relatively stable throughout the pregnancy. The mean FIB levels during the three trimesters of pregnancy were 75.7 ± 43.9 (25–148), 67.6 ± 33.7 (22–146), and 77.9 ± 29.2 (32–148) mg/dl, respectively. Twelve full-term deliveries were achieved using FIB replacement therapy only on the group of labor or cesarean deliveries. The postpartum courses were unremarkable without hemorrhagic or thrombotic complications. Five out of twelve neonates were diagnosed with low levels of FIB in 6 weeks after birth. The pregnancies were uneventful with no signs of bleeding in these congenital hypofibrinogenemia women. Vaginal delivery, instead of cesarean section, may be the superior choice. Successful maternal and neonatal outcomes could be achieved by accurate monitoring of the FIB levels and adequate supportive therapy.

## Introduction

Congenital hypofibrinogenemia, first reported in 1935 [1], is defined as plasmafibrinogen levels below 150 mg/dl [2]. The incidence of the disease is unknown as it is a hereditary bleeding disorder and often caused by heterozygosity of a fibrinogen (FIB) gene mutation [2]. Individuals with plasma FIB concentrations 50–150 mg/dl, considered as having moderate hypofibrinogenemia, are mainly asymptomatic and essentially experience posttraumatic hemorrhages [2,3]. Pregnancy with congenital hypofibrinogenemia is a rare but a high-risk condition. Currently, only a few reports are available on pregnancy associated with hypofibrinogenemia; since 1961, only 10 cases of this disorder during pregnancy have been described [4–11]. Owing to the rarity of the disease and the absences of randomized controlled studies, management of pregnancy with hypofibrinogenemia is challenging. Most clinicians care only a few patients, and hence, their experience is limited.

During the past 5 years, the center of diagnosis and treatment for the hematological systemic disorder associated with pregnancy, in Peking University People's Hospital, experienced 11 cases and 12 successful deliveries with hypofibrinogenemia. In the present article, we described these patients’ plasma levels of FIB throughout pregnancy and summarized the maternal and fetal outcomes.

## Materials and methods

The present retrospective study recruited 11 cases of congenital hypofibrinogenemia in the unit of Peking University people's Hospital during a 5-year period (01/10/2011–30/09/2016). The demographic baseline data, clinical characteristics, laboratory test results, management during pregnancy, mode of delivery, obstetrical complications, as well as, neonatal weight and viability of newborns were collected from the electronic and article records of women with hypofibrinogenemia during pregnancy. The FIB levels and signs of bleeding in the newborns were assessed by telephonic interview 6 weeks after birth. The study was approved by the local Ethics Committee of Peking University people's Hospital. Informed consent was not required as it is a retrospective study. However, all women attending our center were requested to sign an informed consent to allow the usage of their data for scientific purposes and contact for follow-up.

The diagnosis of hypofibrinogenemia was based on a standard hemostasis assessment confirmed by hematologists. As described previously, women with plasma FIB concentration less than 150 mg/dl were enrolled in the study. All the patients had undergone liver function tests to exclude liver disease, as it was a common cause of acquired FIB abnormality.

For patients diagnosed with hypofibrinogenemia, Clauss FIB assays were performed at least once a month, before and after the treatment throughout pregnancy. The signs for bleeding disorders, placental abruption, and fetal growth were monitored during the gestation period. The timing and mode of delivery were decided based on the condition of maternal and fetal complications. We attempted to maintain the full-term pregnancy, if no complications were revealed.

The decision of whether or not to start FIB substitution was based on the patient's personal and family history. At our center, in all cases of pregnancies involving patients with low levels of FIB, hematologists trained in the care of patients with rare bleeding disorders would be contacted. The amount of FIB concentrate (Shanghai RAAS Blood products Co., Ltd, Shanghai, China) was administrated = [target fibrinogen levels (g/l) − measured fibrinogen level] × 0.043 × weight (kg). However, the dosage of the prophylaxis was adapted to the patient's clinical course.

Complications including bleeding in early gestations, premature delivery, fetal growth restriction, placental abruption, postpartum hemorrhage, and postpartum thrombosis were recorded. Postpartum hemorrhage was defined as more than 500-ml vaginal or 1000-ml cesarean blood loss, respectively.

## Results

### Patient characteristics

We encountered 11 cases and 12 full-term deliveries with congenital hypofibrinogenemia in the last 5 years. Four patients were referred to our hospital during the third trimester because of low levels of FIB. The demographic characteristics of the study population are presented in Table 1. The mean age of the 11 patients was 27.1 ± 3.3 (22–32) years. One woman (case 4) was diagnosed with hypofibrinogenemia at the age of 21 following induced abortion with her first pregnancy. Case 6 was diagnosed with hypofibrinogenemia 2 years ago during preoperative coagulation tests for appendectomy. In other cases, hypofibrinogenemia was diagnosed by routine test in progestation and in early pregnancy, and none of them showed bleeding tendency during pregnancy. The results of the clotting study, including prothrombin time (PT), activated partial thromboplastin time, and platelet counts, were normal, with the exception of FIB levels. Their prepregnancy FIB levels were ranged from 48 to 111 mg/dl (Table 1). All patients were excluded for the disease caused by secondary FIB decrease, thereby rendering10 nulliparous cases and one parous case. Positive family histories of hypofibrinogenemia were recorded for 8/11 cases, whereas none of their families had any episodes of hemorrhage or thrombosis. One patient (case 8) was diagnosed with severe hypofibrinogenemia (FIB < 50 mg/dl), whereas the others presented a moderate disease (FIB 50–150 mg/dl).

### Maternal and fetal outcomes

The course of pregnancy was uneventful in the 12 deliveries. The mean gestational age was 39 (38–40) weeks. There were eight vaginal deliveries including one forceps delivery and four cesarean sections. The surgeries were obligatory owing to a scarred uterus, suspected fetal macrosomia, prolonged second stage of labor, and low placenta, respectively. Case 4 experienced a vaginal delivery. She was first presented in our clinic, before 2 years, during her second pregnancy. Clotting tests revealed the trough levels of hypofibrinogenemia as 48 mg/dl. She delivered at 40-week gestation by obstetric forceps because of the meconium-stained amniotic fluid (case 4-1). Then, in her third pregnancy, the plasma FIB level was approximately 48–66 mg/dl throughout the pregnancy; a healthy infant was delivered vaginally (case 4-2).

The birth weight and Apgar scores at 1-min postbirth were reported in all patients. The mean birth weight was 3349 (3000–3780) g. None of the newborns achieved an Apgar score of less than 10 at the first 1 min. A total of 5/12 newborns were diagnosed with low levels of FIB in the follow-up periods (Table 2); however, no bleeding complications were reported.

### Fibrinogen levels throughout pregnancy

FIB levels were supervised closely and regularly during the entire duration of pregnancy. The mean FIB levels during the three trimesters of pregnancy were 75.7 ± 43.9 (25–148), 67.6 ± 33.7 (22–146), and 77.9 ± 29.2 (32–148) mg/dl, respectively (Table 1).

The trough and peak levels of plasma FIB throughout pregnancy are presented in Table 2. One patient (case 8) experienced severe hypofibrinogenemia (trough FIB level was 22 mg/dl). However, no vaginal bleeding was reported during the pregnancy and yet a good pregnancy outcome after FIB substitution was observed.

**Fig. 1:**
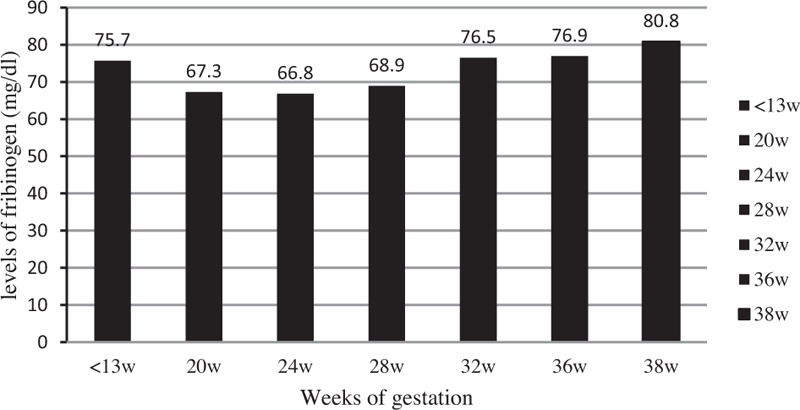
The plasma fibrinogen levels by gestational age.

The plasma FIB levels by gestational age are summarized in Fig. 1. The mean concentrations of FIB were 75.7 ± 43.9 (25–148) mg/dl before 13 weeks of gestation, 67.3 ± 35.6 (28–140) mg/dl in 20 weeks, 66.8 ± 33.4 (35–142) mg/dl in 24 weeks, 68.9 ± 36.4 (22–146) mg/dl in 28 weeks, 76.5 ± 33.9 (32–140) mg/dl in 32 weeks, 76.9 ± 28.5 (48–142) mg/dl in 36 weeks, and 80.8 ± 29.2 (39–148) mg/dl in 38 weeks of gestation.

The mean plasma FIB levels before and after FIB substitutions were 82.4 ± 28.6 and 152.9 ± 42.1 mg/dl, respectively. In the postpartum period, the mean FIB concentrations were then decreased to 118.8 ± 57.0 mg/dl (Table 2).

### Complications

All the patients were followed up in our high-risk pregnancy clinic unit during the duration of pregnancy. No signs of placental abruption or vaginal bleeding were observed during pregnancy. None of the patients suffered from preterm delivery, and the mean gestational age was 39 (38–40) weeks. Any signs of intrauterine growth restriction were not presented, and the average birth weight was 3349 (3000–3780) g. The postpartum courses were unremarkable with no significant hemorrhagic or thrombotic complications (Table 1).

## Discussion

In the present report, 11 pregnant women with congenital hypofibrinogenemia were asymptomatic during the gestation and were diagnosed by routine examination. After examining the obstetric courses, we found that their pregnancies were uneventful and no significant associated bleeding or thrombotic complications were exhibited. The levels of FIB did not decrease significantly as gestational stage progressed. By careful monitoring of the FIB levels and supportive therapy, we achieved 12 term deliveries with 12 healthy newborns.

Congenital hypofibrinogenemia is a rare condition. The previous studies showed that individuals with this disorder are usually asymptomatic as moderate FIB levels are sufficient to prevent spontaneous bleeding [3,12]. A retrospective study including 100 patients with FIB deficiencies indicated that the mean incidence of bleeding episodes was 0.5%, and hypofibrinogenemia with bleeding was nonspontaneous and induced by trauma or surgery [12]. Pregnancy and delivery are extremely high-risk situations; women with hypofibrinogenemia are more prone to bleeding. However, none of the patients in our study showed a bleeding tendency during pregnancy. Although the levels of FIB were 22–48 mg/dl in case 8, no bleeding incident was observed in the periods of gestation.

According to the results of the current study, the amounts of FIB in our patients remained relatively stable during pregnancy. In fact, normal pregnancy is a condition in which significant changes occur in the hemostatic system with an increased tendency to coagulation. The view of the literature revealed that normal pregnancy causes a maternal physiological hypercoagulable state, and FIB levels progressively increase throughout the pregnancy. The study by Liu *et al.*[13] on normal pregnant women demonstrated that the FIB levels increased significantly between weeks of 12 and 20, remained relatively stable between weeks of 21 and 27, started to significantly increase again between weeks of 28 and 35, and remained unchanged between weeks of 26 and 42. Their patients were healthy women, whereas women involved in ours were all with hypofibrinogenemia, which might explicate the FIB levels in our patients were not remarkably higher during the late gestational weeks as compared with early pregnancy. The increasing levels of FIB are considered to prepare for controlling bleeding quickly and effectively. Therefore, pregnant women with low FIB must be taken seriously.

FIB abnormalities have been implicated in many adverse pregnancy outcomes, mainly placental abruption, postpartum hemorrhage, and thrombosis [9]. FIB plays a fundamental role in maintaining the integrity of the placenta by supporting cytotrophoblast spreading for the development of fetal–maternal vascularization [6]. However, in this report, none of the women suffered from significant postpartum hemorrhage or placenta abruption events. This satisfying outcome may be associated with the FIB replacement therapy before delivery. An increased prevalence of placental abruption has been reported in women with hypofibrinogenemia who did not receive FIB replacement [6].

FIB substitution is speculated as the cornerstone to a successful outcome in pregnant women with congenital hypofibrinogenemia [14,15]. Current FIB replacement is administered in the forms of fresh frozen plasma (FFP), cryoprecipitate, or FIB concentrate. Almost all the cases in our report were administered FIB concentrate, and 7/12 deliveries were infused with additional FFP before delivery. Though FFP and cryoprecipitate are less expensive, they require administration of larger volumes, which is of concern in the peripartum period, as women in labor often receive other treatments (e.g., oxytocin) that predispose to fluid overload and edema. Finally, anaphylatoxins in FFP and cryoprecipitate could lead to severe allergic reactions [14]. Thus, with respect to the advantages, FIB concentrate seems to be the best choice.

To evaluate the amount of FIB to be administered, a consensus on the optimal peripartum management is absent. In an open-label prospective study, FIB concentrates leading to a median fibrinogenemia levels of 145 mg/dl were effective in 11 surgical procedures [16]. However, extrapolations from perioperative management to peripartum management may be controversial. Several experts suggest substitution with FIB to attempt a trough level more than 50 mg/dl during the first two trimesters and more than 100 mg/dl at the end of the pregnancy. At delivery, the target FIB levels should be above 200 mg/dl to decrease the risk of postpartum hemorrhage [17,18].

Thrombosis related to FIB substitution has been reported, and an evaluation of thrombotic risk factors should be assessed in pregnant women with FIB disorders [10,19,20]. Placenta abruption is also alleged as a thrombotic complication. One of the potential explanations for thrombotic tendency is that, in the absence of FIB, the antithrombin function of fibrin is impaired. Kaparou *et al.*[10] also suggested that the complication of thrombosis was associated with the coexistence of a structural defect in the FIB molecule. Thromboprophylaxis with low-molecular heparin is recommended by some studies due to the ability to monitor the PT [20]. However, the correlation between FIB replacement and the incidence of thrombosis is still difficult to establish. In a systematic review, Bornikova *et al.*[21] reported two patients with thrombotic complications temporally associated with FIB perfusion; nevertheless, the correlation was not found in the other four cases. As none of the women in the present report showed a personal history of previous thrombotic events and substitution of FIB was stopped in the postpartum period, heparin was not used for treatment. None of them had suffered from any episodes of thrombosis.

Pregnant women with these diseases have been reported previously, most of whom experienced an elective or emergency cesarean delivery. However, we reported seven cases with successful vaginal deliveries including a case delivering two infants. Furthermore, the reasons for surgical deliveries for the remaining four cases were not because of FIB disorders but rather obstetric indications such as stagnation. This phenomenon strongly suggested that pregnancy combined with hypofibrinogenemia could have successful vaginal deliveries under meticulous monitoring and supportive FIB replacement.

## Conclusion

In summary, we postulate that specific treatment, other than close observation and monitoring, is not required if bleeding or thrombotic events were absent in the patient's personal or family history: an appropriate supervision of the coagulation function and focusing on the changes such as FIB and other indexes as necessary. Cesarean delivery is not the indication in women with abnormal FIB levels; on the contrary, vaginal delivery is more beneficial for this subtype.

## Acknowledgements

We thank the participating pregnant women for their conscientious cooperation and the experts at the Division of Hematology for their assistance.

Contributors: H.C. drafted article. M.L. contributed to study concept. J.Y. undertook data collection. X.Z. contributed the revision of article.

### Conflicts of interest

There are no conflicts of interest.

## Figures and Tables

**Table 1:** Summary of 11 cases with congenital hypofibrinogenemia

Characteristics	Number
Numbers of women	11
Numbers of deliveries	12
Age at pregnancy (years)	27.1 ± 3.3 (22–32)
Parity	
Nulliparous	10
Para 1	1
Family history	
Positive	8
Negative	3
FIB levels (mg/dl)	
Prepregnancy	72.6 ± 19.6 (48–111)
1st trimester	75.7 ± 43.9 (25–148)
2nd trimester	67.6 ± 33.7 (22–146)
3rd trimester	77.9 ± 29.2 (32–148)
Gestational age at delivery (weeks)	39 (38–40)
Mode of delivery	
VD	7
FD	1
CS	4
Obstetrical complications	
Bleeding in early gestations	0
Placental abruption	0
Postpartum hemorrhage	0
Postpartum thrombosis	0

Values are given as mean ± SD or numbers. CS, caesarean of gestation; FD, forceps delivery; FIB, fibrinogen; VD, vaginal delivery.

**Table 2:** Fibrinogen levels throughout the pregnancy and obstetric courses among patients with congenital hypofibrinogenemia (*n* = 11)

										Newborns		
Case	Age	Weeks of gestation	FIB (during pregnancy trough–peak (mg/dl)	FIB (intrapartum before treatment) (mg/dl)	Mode of delivery	FIB replacement (g)	Blood loss (ml)	FIB (intrapartum after treatment) (mg/dl)	FIB (1st day of postpartum) (mg/dl)	Birth weight (g)	Apgar score	FIB levels (mg/dl)
1	29	39 + 1	43–83	57	CS (scarred uterus)	FC:6 g	600	168	118	3600	10	39
2	28	40 + 2	67–100	99	CS (marcrosomia)	FC:4 g + FFP:400 ml	500	189	138	3780	10	261
3	26	39 + 1	52–61	60	VD	FC:2 g	515	79	67	3510	10	247
4-1	22	40 + 1	48–80	75	FD	FC:3 g + FFP:400 ml	400	141	105	3530	10	255
4-2	24	38 + 3	50–60	60	VD	FC:4 g + FFP:400 ml	110	123	90	3000	10	214
5	32	40 + 2	140–148	140	VD	FFP:400 ml	50	254	199	3620	10	97
6	28	39 + 3	70–85	80	VD	FC:2 g	300	156	254	3020	10	40
7	27	39	70–96	95	VD	FC:2 g	100	140	123	3110	10	155
8	32	38 + 5	22–48	37	VD	FC:4 g + FFP:400 ml	150	142	58	3060	10	165
9	22	39 + 2	87–124	94	CS (labor stagnation)	FC:2 g + FFP:400 ml	400	163	66	3370	10	43
10	26	38	95–111	109	CS (placenta lower)	FC:2 g	400	156	104	3200	10	29
11	29	40 + 2	65–95	73	VD	FC:2 g + FFP:400 ml	100	124	103	3390	10	327

Apgar score is expressed at 1 min. CS, caesarean of gestation; FC, fibrinogen concentrate; FD, forceps delivery; FFP, fresh frozen plasma; FIB, fibrinogen; VD, vaginal delivery.
